# Lysine-Directed Post-translational Modifications of Tau Protein in Alzheimer's Disease and Related Tauopathies

**DOI:** 10.3389/fmolb.2017.00056

**Published:** 2017-08-11

**Authors:** Christiana Kontaxi, Pedro Piccardo, Andrew C. Gill

**Affiliations:** Roslin Institute and Royal (Dick) School of Veterinary Sciences, University of Edinburgh Edinburgh, United Kingdom

**Keywords:** tau, acetylation, methylation, ubiquitylation, SUMOylation, glycation, paired helical filaments, tauopathies

## Abstract

Tau is a microtubule-associated protein responsible mainly for stabilizing the neuronal microtubule network in the brain. Under normal conditions, tau is highly soluble and adopts an “unfolded” conformation. However, it undergoes conformational changes resulting in a less soluble form with weakened microtubule stabilizing properties. Altered tau forms characteristic pathogenic inclusions in Alzheimer's disease and related tauopathies. Although, tau hyperphosphorylation is widely considered to be the major trigger of tau malfunction, tau undergoes several post-translational modifications at lysine residues including acetylation, methylation, ubiquitylation, SUMOylation, and glycation. We are only beginning to define the site-specific impact of each type of lysine modification on tau biology as well as the possible interplay between them, but, like phosphorylation, these modifications are likely to play critical roles in tau's normal and pathobiology. This review summarizes the latest findings focusing on lysine post-translational modifications that occur at both endogenous tau protein and pathological tau forms in AD and other tauopathies. In addition, it highlights the significance of a site-dependent approach of studying tau post-translational modifications under normal and pathological conditions.

## Introduction

Neurodegenerative diseases of the central nervous system are characterized by selective loss of synapses and neurons, glial activation, progressive irreversible neural dysfunction, cognitive impairment and eventually death (Verkhratsky et al., 2014; Kovacs, 2016). Many neurodegenerative diseases are also known as conformational diseases- or proteinopathies-due to the presence of pathological forms of proteins that accumulate and deposit in the brain (Carrell and Lomas, 1997). For this reason, it has been assumed that the aggregation of misfolded proteins is the molecular cause of neurodegeneration. Proteins participating in aggregates lack their normal tertiary structure and, hence, they are incapable of serving their typical functions in living cells. The aberrantly-folded forms may also acquire a novel, neurotoxic role (Ballatore et al., 2007). A common class of neurodegenerative diseases includes the disorders associated with the filamentous inclusions of tau aggregates in nerve cells and glia, which are known collectively as tauopathies (Spillantini et al., 1997; Ferrer et al., 2014). One of the main pathological hallmarks of Alzheimer's disease (AD) is the intraneuronal accumulation of neurofibrillary tangles (NFTs) consisting of misfolded tau protein and, therefore, AD is considered to be partly a tauopathy and is one of the most widely studied. Other tauopathies include progressive supranuclear palsy, frontotemporal dementia with parkinsonism-17, corticobasal degeneration, argyrophilic grain disease, Pick's disease and Huntington's disease (Hernandez and Avila, 2007; Gratuze et al., 2016).

**Figure 1:**
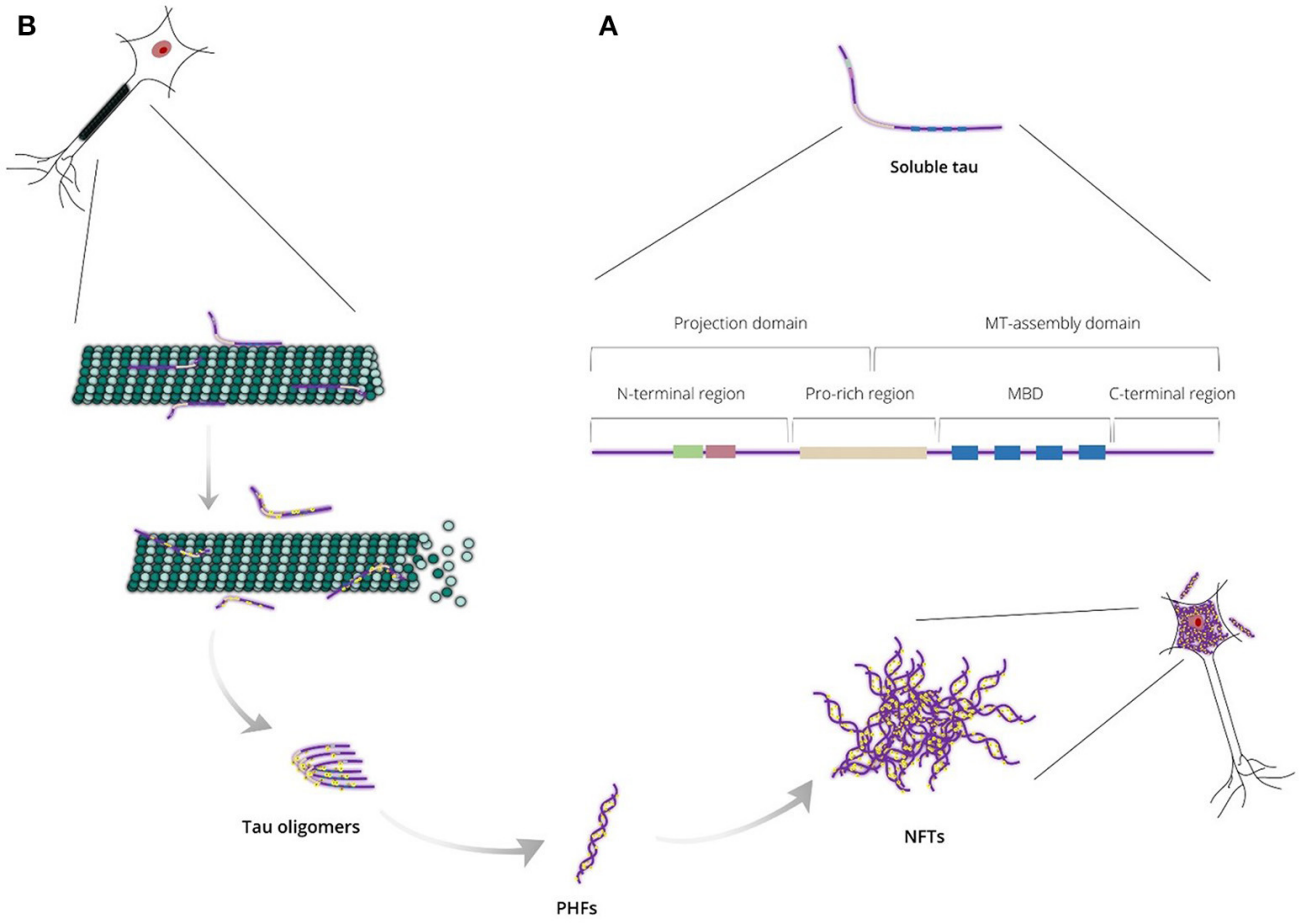
**(A)** Endogenous tau is a highly soluble protein adopting an “unfolded” conformational state and consisting of two major domains: the projection domain that projects from microtubules and the microtubule (MT)-assembly domain that interacts with tubulin heterodimers. Tau can also be divided into four regions: the N-terminal region, the proline-rich (Pro-rich) region, the microtubule-binding domain (MBD) and the C-terminal region. **(B)** Tau is responsible mainly for stabilizing neuronal microtubules based on its phosphorylation state. Abnormal tau hyperphosphorylation weakens tau affinity for microtubules and, thus, releases tau in the cytosol and destabilizes the microtubule bundles. Unbound hyperphosphorylated tau assembles into pathogenic inclusions that deposit in brain tissues causing pathological phenotypes.

Tau protein is a microtubule-associated protein (MAP) expressed abundantly in neurons and, to a lesser extent, in astrocytes and oligodendrocytes. In neurons, tau localizes predominantly to the axonal cytoplasm, but it can also be found in the nucleus and dendrites (Binder et al., 1985; Migheli et al., 1988; Loomis et al., 1990). Tau is responsible mainly for microtubule assembly and stabilization, thus maintaining the normal morphology of the neuronal cells and enabling axonal transport. Tau may also be involved in other activities such as neurogenesis and iron export (Lei et al., 2012; Pallas-Bazarra et al., 2016). It is encoded by the *MAPT* gene, which includes 16 exons located on the human chromosome 17 (Neve et al., 1986; Lee et al., 1988). Based on protein structure, tau can be divided into four regions: (i) a N-terminal projection region that protrudes from the microtubules to which tau is bound and is responsible for interacting with other, non-microtubular partners; (ii) a proline-rich region that contains seven PXXP motifs, which serve as binding sites for signaling proteins; (iii) a microtubule-binding domain (MBD) that contains three or four repeat regions, R1, R2, R3, and R4, which are essential for binding to microtubules through their conserved KXGS motifs, and the flanking regions between them; (iv) a C-terminal region (Figure 1A; Mandelkow et al., 1995). There is heterogeneity at the level of transcription due to alternative splicing of exon 10, resulting in the generation of two different tau isoforms; these isoforms are known as 3R or 4R depending on whether they contain three or four repeat regions within the MBD (Lee et al., 1989).

Under normal conditions, tau is highly soluble and is classed as an intrinsically disordered protein. It is believed to have little tendency for aggregation in this intrinsically disordered state (Schweers et al., 1994; Mukrasch et al., 2009), however, tau can undergo conformational changes resulting in altered physical and chemical properties, including a decrease of solubility. Thereby, conformationally-stable insoluble structures are formed as tau assembles into higher-polymerized aggregates that differ between different tauopathies (Yoshida, 2006). In AD, tau lesions include neurofibrillary tangles (NFTs) in neuronal cell bodies, neuropil threads in neurites and dystrophic neurites in neuritic plaques; in electron microscopy, tau assemblies form mainly paired helical filaments (PHFs) that consist of both 3R and 4R isoforms (Figure 1B). In contrast, 3R or 4R tau isoforms are preferentially accumulated in a variety of tauopathies, such as progressive supranuclear palsy and Pick's disease (Dickson et al., 2011). The processes underpinning the formation of tau aggregates are not completely understood, but appear to involve a variety of post-translational modifications occurring at many sites throughout tau, including phosphorylation, O-Glc-NAcylation, glycation, nitration, acetylation, methylation, SUMOylation, ubiquitylation, oxidation, and truncation (Martin et al., 2011).

Since the phosphorylation state of tau controls its intrinsic affinity for microtubules and given the fact that aggregated tau species have been shown to be hyperphosphorylated at several serine, threonine and tyrosine residues, numerous studies have focused on exploring tau phosphorylation (Grundke-Iqbal et al., 1986; Williamson et al., 2002). Conversely, tau modifications that extend to lysine residues have not yet been analyzed as extensively, but it is likely that they might be as important as phosphorylation in dictating the biophysical properties of tau, because they profoundly alter the charge of the protein. This review gives an overview of the latest findings concerning the lysine-directed tau post-translational modifications, which include acetylation, methylation, ubiquitylation, SUMOylation and glycation, and discusses the impact on tau biology of the possible cross-talk between them. It also emphasizes the need to achieve a complete understanding of the biological role of lysine site-specific modifications in both endogenous and aggregated tau, in order to shed light on the molecular events underlying the pathological transition of tau that characterizes tau-mediated neurodegeneration.

## Tau acetylation

**Figure 2:**
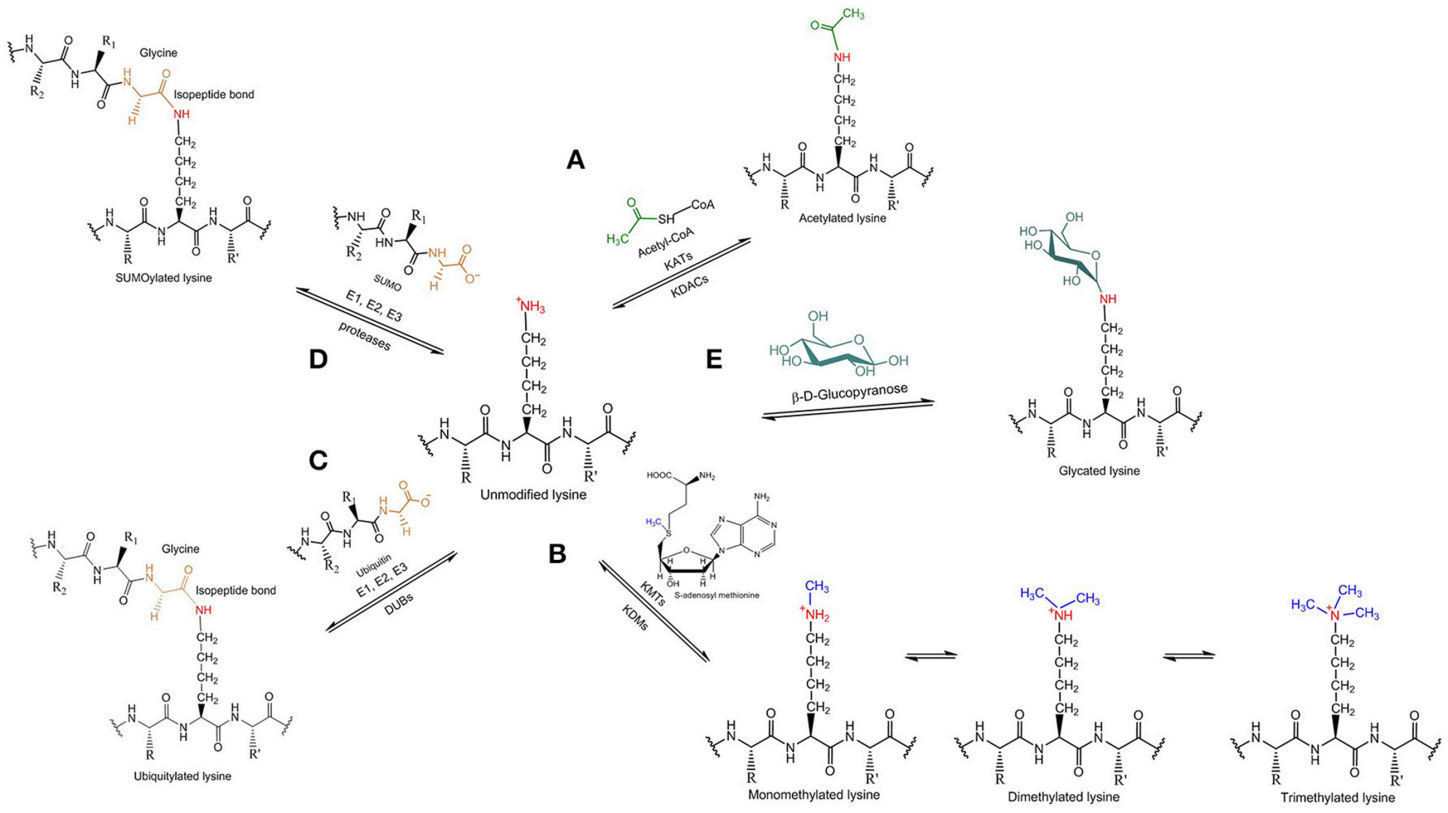
Tau lysine-specific post-translational modifications include **(A)** acetylation, **(B)** methylation, **(C)** ubiquitylation, **(D)** SUMOylation, and **(E)** glycation.

Acetylation is a co- or post-translational modification that is best known for modifying the N-termini of eukaryotic proteins (around 85% of human proteins are believed to be N-terminally acetylated) as well as for modifying the side chains of specific lysine residues in histones, thereby altering chromatin structure and providing epigenetic control of transcription. As a result, the enzymes responsible for acetylating and deacetylating protein substrates at lysine residues are called histone acetyltransferases and histone deacetylases, respectively, but given the fact that a variety of other proteins except for histones can be acetylated the terms lysine acetyltransferase (KAT) and lysine deacetylase (KDAC) are more precise. The source of the acetyl group in protein acetylation reactions is acetyl-CoA and it has been demonstrated recently by Min et al. (2010), that lysine side chains of tau protein can be acetylated (Figure 2A). NMR analysis of recombinant human tau acetylated enzymatically *in vitro* has shown that tau displays an overall acetylation level of 6 ± 2 acetyl groups per molecule (Kamah et al., 2014). Tau can be acetylated by either the protein p300 or the CBP acetyltransferase, a CREB-binding protein and close homolog of the p300 acetyltransferase, and deacetylated by the NAD^+^-dependent sirtuin 1 deacetylase (SIRT1; Min et al., 2010). A decrease in the levels of both SIRT1 mRNA and protein has been associated with enhanced PHF-tau accumulation in AD patients (Julien et al., 2009), thus indicating a negative correlation between the regulation of SIRT1 and tau accumulation. Moreover, histone deacetylase 6 (HDAC6), located mainly in the cytoplasm, has been shown to interact with tau in the MBD (Ding et al., 2008) suggesting that HDAC6 is another possible tau deacetylase, and Cook et al. found that HDAC6 could deacetylate tau on KXGS motifs (Cook et al., 2014). HDAC6 has also been shown to be involved in assisting the clearance of misfolded huntingtin through autophagic degradation by mediating the retrograde transport of autophagic components to huntingtin aggresomes (Iwata et al., 2005), supporting the notion that HDAC6 might act to protect neurons against abnormal tau rather than act only as a tau the deacetylase. In contrast to HDAC6, both p300 and SIRT1 are localized mainly in the nucleus (Michishita et al., 2005; Blanco-Garcia et al., 2009), whereas tau resides predominantly in the axonal cytoplasm (Binder et al., 1985); if they are to regulate tau acetylation exclusively and directly then the mechanisms underpinning the migration of these deacetylases to the cytosolic compartment of adult neurons need to be established.

Remarkably, tau also has intrinsic acetyltransferase activity and, hence, can catalyze its own acetylation by using cysteine residues in the MBD—C291 and C322 (all amino acid numbers in the manuscript refer to human tau) in the R2 and R3 repeats, respectively—as intermediates to enable transfer of the acetyl group from acetyl-CoA to certain lysine residues both intra- and inter-molecularly (Cohen et al., 2013). Different tau isoforms may have different tendency to undergo autoacetylation, with 4R isoforms displaying higher levels of autoacetylation compared to 3R isoforms, which lack the R2 repeat that includes C291 hence contain only the C322 residue (Cohen et al., 2016). Despite the intrinsically disordered nature of tau, both C291 and C322 residues participate in α-helical structures, which ensures a relatively ordered conformation and brings cysteine and lysine residues (C291 and K274, C322 and K340) into close proximity for the chemical reaction(s) to take place (Luo Y. et al., 2014). Since acetylated tau is unable to bind to microtubules (Cohen et al., 2011), it has been suggested that tau autoacetylation might serve as an autoinhibitory mechanism to prevent interaction between tau and microtubules (Cohen et al., 2013). Tau self-acetylation has also been shown to induce tau autoproteolysis, catalyzed by the same cysteine residues, during which about 17 kDa and 12 kDa C- and N-terminal fragments, respectively, are generated (Cohen et al., 2016). Mass spectrometry analysis of the N-terminal end of each fragment identified that the putative autocleavage sites on tau are located within the R2 and R4 repeats (Cohen et al., 2016).

**Figure 3:**
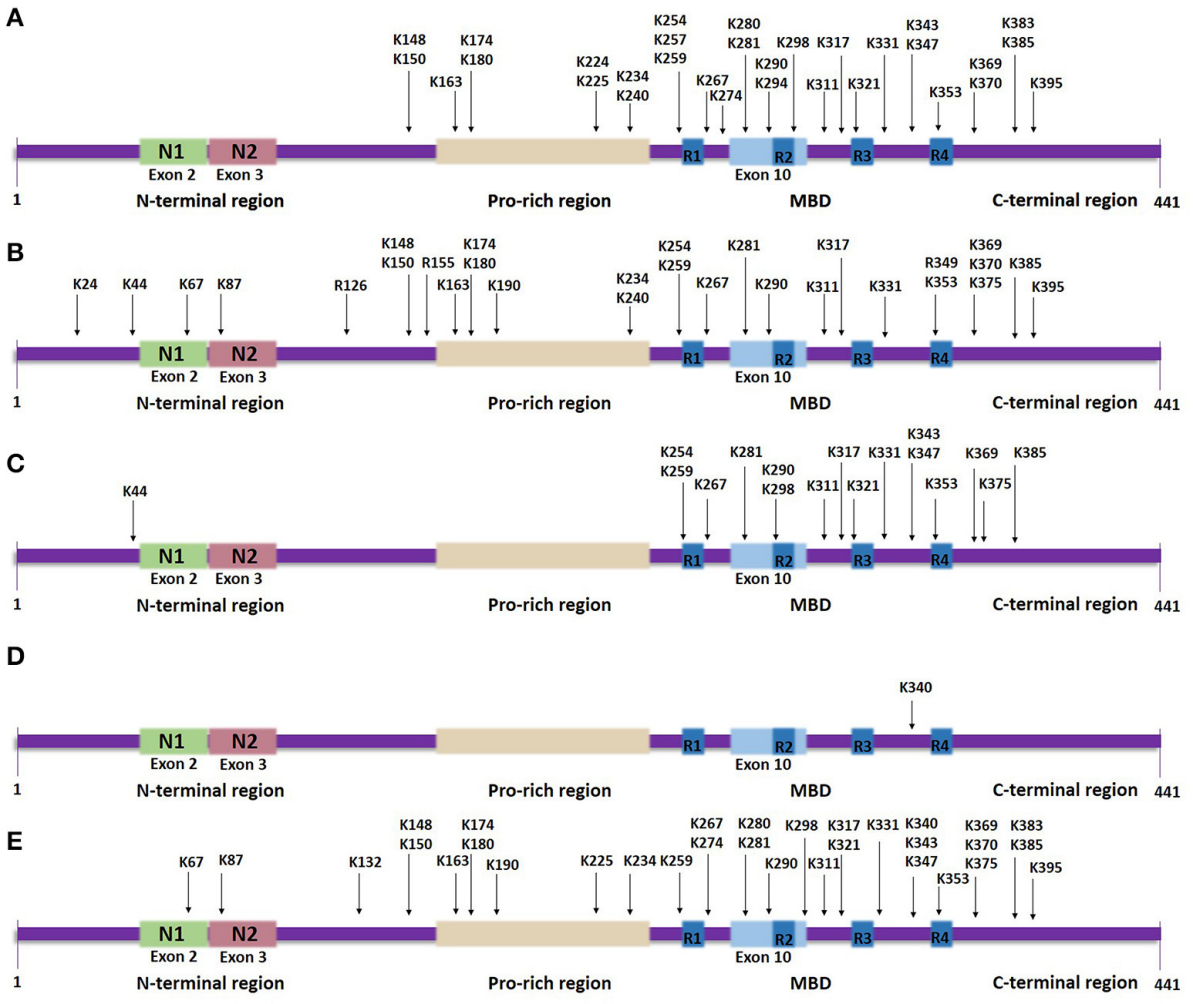
Tau lysine-specific sites that are modified post-translationally based on the longest human tau isoform. The modified sites include the total number of sites that have been identified both *in vivo* and in vitro, in normal tau as well as in pathological states of tau. **(A)** Tau acetylation sites. **(B)** Tau methylation sites. **(C)** Tau ubiquitylation sites. **(D)** Tau SUMOylation site. **(E)** Tau glycation sites.

Most lysine residues that are putative sites of acetylation are distributed in the MBD, whilst, a few are found in the N-terminal and C-terminal regions (Figure 3A). Proteomic studies revealed 23 lysine residues throughout the tau sequence that can potentially be acetylated by p300 *in vitro*, of which 13 lysines are found in the MBD (Min et al., 2010). NMR analysis assigned several lysine residues as potential CBP-catalyzed acetylation sites in accordance with previous mass spectrometric data, including two novel highly acetylated lysines, K240 and K294 (Kamah et al., 2014). Acetylation of K163, K174 and K180, located in the proline-rich region, has also been confirmed *in vivo* (Min et al., 2010). A further study detected K163 in the proline-rich region, K280 and K281 in the second repeat region and K369 in the MBD as the major sites of tau acetylation (Cohen et al., 2011). Recently, 13 putative lysine sites were discovered *in vivo* including K343 and K347 in the MBD (Morris et al., 2015)—acetylation in this region could affect the affinity of tau for microtubules.

Of the putative acetylation sites, K174, K274, K280, and K281 have received most attention concerning their significance in regulating tau function. Acetylation of K280 is of particular interest since it has been suggested to be a critical event for the formation of pathogenic tau based on data showing that this modification decreases microtubule binding and, also, precedes and possibly enhances tau fibrillization into PHFs (Irwin et al., 2012). Moreover, transgenic Drosophila models overexpressing a mutant form of human tau (K280Q) to mimic acetylation show increased phosphorylation at S262 and T212/S214 and enhanced tau levels, possibly due to either increased oligomerization or differential protein degradation, thus suggesting that acetylated K280 contributes to pathological events underlying tau toxicity (Gorsky et al., 2016). Conversely, examining the co-localization of acetylated K280 immunoreactivity with multiple tau epitopes in AD revealed a possible sequence of events, according to which tau hyperphosphorylation occurs before tau acetylation at K280, which is then followed by subsequent tau truncation (Irwin et al., 2012). Notably, immunohistopathological studies revealed that in normal brains K280 is not identified to be acetylated, whereas it is predominantly detected in NFTs and, to a lesser extent, in neuropil threads or pretangles (Cohen et al., 2011; Irwin et al., 2012). Acetylated K280 is detected in neuronal and glial inclusions in many tauopathies, such as argyrophilic grain disease, sporadic and familial AD, frontotemporal dementia with parkinsonism-17, corticobasal degeneration and progressive supranuclear palsy (Cohen et al., 2011; Irwin et al., 2012, 2013). However, Pick's disease is a 3R tauopathy and 3R tau isoforms lack exon 10, where K280 is located, so that a low degree of acetylated K280 was found only in a subset of 4R tau containing lesions (Irwin et al., 2013). This indicates that acetylation of K280 is not sufficient for causing tau-induced neurotoxicity.

In contrast to K280, acetylated K274, located in the first repeat region, was shown to be present in neuronal and glial inclusions of both 3R and 4R human tauopathies from a variety of sporadic as well as familial cases, with the exception of the 4R tauopathy argyrophilic grain disease (Grinberg et al., 2013). Pathological acetylation of K274, as well as K281, has been reported to cause downregulation of the cytoskeletal protein network of the axon initial segment leading to destabilization of the microtubule barrier in this region and consequent somatodendritic mis-sorting of tau, which represents an early event of neurodegeneration (Sohn et al., 2016). Apart from enabling tau mislocalization, these modifications have been associated with synaptic dysfunction and memory deficits observed in AD brains (Tracy et al., 2016) with possible mechanisms involving the kidney/brain protein, a postsynaptic memory-associated protein. Specifically, primary hippocampal neurons expressing K274/K281-specific acetylation-mimic mutants failed to recruit AMPA receptors to the postsynaptic surface of spines, whilst activity-dependent postsynaptic actin polymerization was disrupted; site-specific acetylated tau was shown to disturb the postsynaptic distribution of kidney/brain protein, which contributes to impaired actin polymerization and trafficking of AMPA receptors in the postsynaptic membrane (Tracy et al., 2016). Lastly, another significant residue that can be acetylated is K174 found in the proline-rich region. Pseudoacetylation at K174 (K174Q) was reported to attenuate tau clearance resulting in increased tau accumulation and was sufficient to induce behavioral deficits *in vivo*, such as memory and learning impairments. Thus, acetylation of K174 is likely to be an additional key modification regulating tau-induced toxicity (Min et al., 2015).

The finding that tau can be acetylated gave rise to further research studies examining the impact of these modifications on tau physiopathology. In general, acetylation that is elevated by cellular stress, such as Aβ accumulation (Min et al., 2010), seems to affect tau biology via two different processes. The first involves the dysregulation of tau homeostasis due to prevention of degradation mediated by the ubiquitin-proteasome system (UPS; Min et al., 2010). Most of the putative acetylated lysine residues that are distributed in the MBD can also be polyubiquitylated (see below) to mark tau for degradation (Morris et al., 2015). Hence, acetylation of lysines can prevent their polyubiquitylation resulting in insufficient turnover of both endogenous and hyperphosphorylated tau (Min et al., 2010). Primary cultured neurons lacking SIRT1 activity that, as a result, show enhanced acetylated tau levels, also have impaired tau turnover (Min et al., 2010). Similarly, inadequate turnover of hyperphosphorylated tau is observed when tau also becomes hyperacetylated (Min et al., 2010). Impaired clearance of tau is believed to be one of the major factors that leads to tau accumulation by increasing the pool of tau available for aggregation by maintaining proteins that should be normally degraded.

The second mechanism by which acetylation is suggested to change tau function is through the impairment of tau-microtubule interactions (Cohen et al., 2011), since acetylation neutralizes the positive charge of lysine residues in the MBD, thereby disabling tau binding to negative charges on the microtubule surface (Luo Y. et al., 2014). Of course, the release of tau from microtubules might act collaboratively with impaired tau degradation in enhancing tau pathological accumulation. Nevertheless, whether acetylation facilitates tau fibrillization remains to be elucidated. However, acetylation of KXGS motifs (K259, K290, K312, and K353) has been reported to prevent tau phosphorylation at these same motifs and decreases aggregation of recombinant tau *in vitro* (Cohen et al., 2011; Cook et al., 2014). Furthermore, KXGS motifs are hypoacetylated in AD brains and in a mouse model of tauopathy (Cook et al., 2014). This discovery implies that acetylation of particularly KXGS motifs is an event possibly occurring merely in normal tau, but its role needs further investigation. In any case, the fact that other MAP proteins, such as MAP2, which share highly conserved repeats with tau, undergo lysine acetylation in the MBD and cysteine-dependent autoacetylation indicates that acetylation might be a conserved regulatory mechanism of MAP activity in governing cytoskeletal dynamics (Hwang et al., 2016).

## Tau methylation

Methylation refers to the enzymatic addition of one or more methyl groups to protein substrates. Typically, the methyl group derives from S-adenosyl methionine and it is added to the terminal amino group of lysine or arginine side chains of the target protein. Depending on the residue that is modified, lysine methyltransferases (KMTs) and protein arginine methyltransferases, respectively, are responsible for methylating the protein substrates; accordingly, the reverse reaction can be catalyzed by several lysine demethylases (KDMs), but no arginine demethylases are yet known. Lysine methylation of tau (Figure 2B) is a relatively recent discovery that, to date, has not received the same attention as acetylation. Mass spectrometric analysis of PHFs derived from AD brains showed that several lysine residues, distributed in the projection domain and MBD of tau protein, can be methylated (Thomas et al., 2012). So far, the specific enzymes involved in tau methylation have not been identified.

Lysine methylation has been detected in tau protein isolated from both pathological and normal brains (Figure 3B). In human AD brains, aggregated tau is monomethylated at seven lysine residues found in the proline-rich region and the R1 and R2 repeats within the MBD, of which K180 and K267 appear to be more frequently methylated, in contrast to K290 that displays the lowest level of methylation (Thomas et al., 2012). Later studies showed that endogenous tau from cognitively-normal human brains can be monomethylated as well as dimethylated at different lysine residues (Funk et al., 2014; Morris et al., 2015). Extracted soluble tau is methylated at up to 11 sites, more than found to be modified in PHF-tau, and which are distributed throughout the sequence, whilst *in vitro* reductive methylation of recombinant tau led to detection of 23 methylated lysines (Funk et al., 2014). Furthermore, not only lysine residues, but the arginine residues R126, R155, and R349 were detected as possible sites of monomethylation in normal mouse tau and in a mouse model of AD (Morris et al., 2015). Tau modified by the addition of three methyl groups at a single site—tri-methylation—has not yet been found in either healthy or pathological states.

Since K254 was found to be mainly methylated and, to a lesser extent, ubiquitylated (see below) in PHF-tau, it has been suggested that methylation may block UPS-mediated degradation of tau leading to a further enhancement of tau levels in the cell (Thomas et al., 2012). At the same time, phosphorylation of S262, which reduces tau affinity for microtubules, is found more frequently in the presence of methylated K267 in PHF-tau (Thomas et al., 2012). This suggests that lysine methylation, apart from preventing degradation by the UPS, might result in abnormal phosphorylation of tau. In addition, most of the potentially methylated sites of PHF-tau are subject to acetylation as well, suggesting that these modifications might compete for the same site-specific lysine residues on pathological tau. K163, K174 and K180 were identified *in vivo* as possible sites of both acetylation and methylation (Min et al., 2010; Thomas et al., 2012), thus signifying the importance to explore the competing factors that may govern tau pathological modifications. However, significant putative acetylated sites, such as K280 and K281, have not yet been detected as being methylated (Thomas et al., 2012).

Immunohistochemical studies using a combination of a polyclonal antibody, that recognizes methylated tau epitopes, and antibodies specific for epitopes present on PHF-tau demonstrated that methylated tau is highly colocalized with NFTs especially in late-stage AD brains (Thomas et al., 2012). Given that the regions found to be potentially methylated in pathological tau are essential for interactions with microtubules and other partners, lysine methylation may serve to suppress tau binding to these partners. Moreover, lysine residues have been shown to participate in electrostatic interactions facilitating abnormal protein aggregation (Sinha et al., 2011) and, as a result, it has been suggested that methylation of lysine residues possibly enables interactions between tau molecules, playing thus an important role in tau self-assembly and NFT formation. This is further supported by the discovery that lysine methylation of other non-histone proteins, such as the transcription factor p53, affects both protein-protein interactions and protein stability (West and Gozani, 2011). In contrast, although the impact of lysine methylation on endogenous tau activity remains unknown, recombinant tau methylated *in vitro* via reductive methylation appeared to have low tendency for aggregation and the modifications actually promoted tubulin assembly (Funk et al., 2014), indicating that methylation of tau might have a protecting role against abnormal aggregation of the protein. However, the specificity of a chemically-induced modification is generally low, making it difficult to interpret its effects on tau. Therefore, an extensive investigation of the site-dependent lysine methylation observed *in vivo* would contribute to the understanding of this modification in tau biology.

## Tau ubiquitylation

Ubiquitylation involves the formation of an isopeptide bond between the C-terminal carboxyl group of the small regulatory protein ubiquitin and the ε-amino group present in lysine side chains of proteins (Figure 2C). Sequential addition of more than one ubiquitin molecule by ubiquitin-chain elongation factors (E4 polyubiquitin ligases) can result in the generation of long polyubiquitin chains, which differ in terms of the specific lysine residue that is used to form an isopeptide bond to the C-terminal glycine of the next ubiquitin molecule defining, therefore, the type of polyubiquitin linkage. In general, it is polyubiquitylation rather than the attachment of a single ubiquitin that acts to induce the proteolytic degradation of targeted proteins in the cytoplasm by the UPS, but different types of polyubiquitin linkage affect the fate of the modified protein in various ways. Whereas, K48-polyubiquitylated proteins are degraded by the UPS pathway with the help of Rad23 proteins, which stimulate their binding to the proteasome (Nathan et al., 2013), K63-polyubiquitylated proteins are directed to the lysosomal-autophagic pathway. K63 polyubiquitin chains were shown to interact selectively with the cellular factor ESCRT0 (*Endosomal Sorting Complex Required for Transport*), which prevents the binding to the 26s proteasome (Nathan et al., 2013), permitting K63-polyubiquitylated proteins to serve different functions, such as receptor endocytosis, intracellular signaling and DNA repair (Mukhopadhyay and Riezman, 2007; Ikeda and Dikic, 2008).

Ubiquitylation takes place in three successive stages, each of them involving a distinct enzyme: an E1 ubiquitin activating enzyme that catalyzes the ATP-dependent ubiquitin activation, an E2 ubiquitin conjugating enzyme onto which activated ubiquitin is transferred through a transesterification reaction and, finally, an E3 ubiquitin ligase essential for catalyzing the formation of the isopeptide bond between ubiquitin's terminal glycine and the target protein. Amongst these, the E3 ubiquitin ligase primarily defines the specific protein substrate that will be ubiquitylated.

### Enzymes regulating tau ubiquitylation

Several E3 ligases have been reported to ubiquitylate tau, including the C-terminus of the Hsc70-interacting protein (CHIP), TNF receptor-associated factor 6 (TRAF6) and axotrophin/MARCH7 (Petrucelli et al., 2004; Babu et al., 2005; Flach et al., 2014), whereas other E3 ubiquitin ligases, such as parkin and Cbl, failed to ubiquitylate hyperphosphorylated tau (Petrucelli et al., 2004; Shimura et al., 2004). CHIP was shown to ubiquitylate tau *in vivo* and *in vitro* by associating with tau's MBD and the nearby proline-rich region, thereby promoting the proteasome-mediated degradation of soluble tau (Petrucelli et al., 2004). CHIP has intrinsic E3 ubiquitin ligase activity via a U-box domain, and ubiquitylates tau through K48 or K63 linkages (Petrucelli et al., 2004). Two types of E2 ubiquitin conjugating enzymes, UbcH5a and Ubc13-Uev1a, have been identified to interact with CHIP, but it is likely that the sequential interaction of Ubc13-Uev1a with other E2 enzymes is responsible particularly for ubiquitylating tau with K63 polyubiquitin chains (Xu et al., 2008). It has also been reported that hyperphosphorylated tau derived from AD brains can be polyubiquitylated by use of HbcH5B as an E2 enzyme (Shimura et al., 2004). CHIP might mediate tau degradation in both physiological and diseased conditions regardless of its phosphorylation state (Zhang et al., 2008), however, tau hyperphosphorylation was shown to be a recognition requirement for ubiquitylation by the CHIP-Hsc70 complex in the presence of HbcH5B (Shimura et al., 2004).

CHIP interacts directly with the molecular chaperone Hsp70/Hsp90 and increased Hsp70 activity in HEK293 cells was found to weaken CHIP activity, thereby suppressing CHIP-mediated tau ubiquitylation; given that Hsp70 interacts with tau this suggests an antagonistic relationship between the two interacting partners (Petrucelli et al., 2004). A quantitative analysis of CHIP in human and mouse brains revealed that the levels of CHIP and Hsp70 appear to be increased in AD compared with normal controls (Sahara et al., 2005). Also, in several human tauopathies, CHIP localized to tau neuronal and glial lesions, with 3R tauopathies displaying more CHIP immunoreactivity than 4R or 3R + 4R tauopathies (Petrucelli et al., 2004). Increased accumulation of aggregated tau species was observed in a cell culture system overexpressing CHIP (Petrucelli et al., 2004). In contrast, insoluble tau accumulation has been increased in a mouse model lacking CHIP (Sahara et al., 2005). These data suggest that the CHIP-Hsp70 complex might be a key molecular assembly affecting tau pathogenic events, but its role needs further investigation.

CHIP may also affect abnormal tau levels indirectly via two possible processes. Firstly, it has been demonstrated that CHIP ubiquitylates and controls the levels of HDAC6, a deacetylase of the molecular chaperone Hsp90, and enhanced levels of HDAC6 have been associated with augmented tau accumulation (Cook et al., 2012). The second pathway involves a different stress-induced substrate of CHIP, the cellular kinase Akt, which prevents CHIP-induced tau ubiquitylation and its subsequent degradation by regulating the CHIP-Hsp90 complex directly, competing with tau for binding to CHIP or promoting tau hyperphosphorylation at S262/S356, a tau species that is not recognized by the CHIP-Hsp90 complex (Dickey et al., 2008).

Another candidate E3 ubiquitin ligase for tau is TRAF6, which ubiquitylates tau via K63 linkages, apparently inducing tau degradation through the UPS pathway (Babu et al., 2005). Although, K63 polyubiquitylation has not been associated with proteolytic degradation of substrates by the UPS, the 26s proteasome is capable of binding and degrading K63-polyubiquitylated proteins *in vitro* in a similarly way to proteins modified by K48-polyubiquitin chains (Hofmann and Pickart, 2001). In aggregates extracted from AD brains TRAF6 was shown to be colocalized with the ubiquitin-associating protein sequestosome 1/p62, a cellular protein responsible for interacting with the proteasomal subunit Rpt1 (Babu et al., 2005). p62 interacts with the K63 polyubiquitin chain of tau through its UBA domain (Babu et al., 2005), so is suggested to be an essential intermediate of TRAF6-mediated tau degradation.

Recently, by use of yeast-two-hybrid systems it was shown that axotrophin/MARCH7 is a tau-interacting protein and, also, has E3 ligase activity via its C-terminal RING-variant domain in the presence of the E2 enzymes UbcH5b/c, UbcH6 and, to a lesser extent, UbcH13 (Flach et al., 2014). Axotrophin is able to induce tau monoubiquitylation *in vitro* with tau isoforms being preferentially modified at multiple sites, including some located in the MBD, which led to weakened tau binding to microtubules (Flach et al., 2014). In AD brain tissues, axotrophin was observed to colocalize with tau aggregates in different cellular compartments, such as the cell soma or dendrites, in contrast to normal brains and tau knockout mice, where axotrophin was found predominantly in the nucleus implying that tau affects the intracellular sorting of axotrophin (Flach et al., 2014).

Ubiquitylation is a reversible process with specific deubiquitinases (DUBs), catalyzing the cleavage of the isopeptide bond. So far, only Otub1, a cysteine protease deubiquitinating enzyme, has been reported to deubiquitinate endogenous tau in mouse brains and prevent tau degradation by removing K48 polyubiquitin chains (Wang et al., 2017). Using primary neurons derived from a tau transgenic mice model, it was shown that Otub1 expression leads to increased total tau levels confirming the role of Otub1 as a tau deubiquitinating enzyme and suggesting that impairment of Otub1 expression could result in impaired tau clearance (Wang et al., 2017). Furthermore, Otub1 was shown to contribute directly to tau pathology, since Otub1 expression in primary neurons leads to enhanced tau aggregation and increased levels of oligomeric tau forms (Wang et al., 2017), which might be important given that tau oligomers have emerged as the pathogenic species in tauopathies (Lasagna-Reeves et al., 2010).

### Putative ubiquitylated sites on tau

Mass spectrometric analysis of soluble PHF-tau immunopurified from AD brains revealed three putative ubiquitylated lysine residues: K254 and K353 located in the R1 and R4 repeat sequences, respectively, and K311 found in the flanking region between the R2 and R3 repeat sequences (Figure 3C; Cripps et al., 2006). K290, another lysine residue located within the MBD, was detected to be ubiquitylated in a mouse model of AD (Morris et al., 2015). Endogenous murine tau is ubiquitylated at 15 possible lysine residues, which are distributed mostly throughout the MBD region, except for K44 that is found in the N-terminal region (Morris et al., 2015); some of these residues are subject to methylation as well. For example, K254 from PHF-tau isolated from late-stage AD regions is either ubiquitylated or methylated, with the latter strongly dominating (Thomas et al., 2012). Moreover, to date all of the lysine residues discovered to be possibly ubiquitylated are sites that could be acetylated as well. These discoveries suggest that some modifications occur at the expense of others indicating even more strongly the importance to clarify the site-specific biochemical cross talk between competing post-translational modifications. Furthermore, mass spectrometric data identified three types of polyubiquitin linkage through which PHF-tau is modified, K6, K11 and, predominantly, K48 linkages (Cripps et al., 2006), whereas soluble tau can also be ubiquitylated via K63 polyubiquitin conjugation (Petrucelli et al., 2004). Notably, coexpression of K63 ubiquitin with a disease-associated tau mutant in SH-SY5Y neuroblastoma cells enhanced the formation of ubiquitin-enriched tau-positive inclusions (Tan et al., 2008).

### The effect of ubiquitylation on tau biology

The studies concerning the role of tau ubiquitylation in its degradation are rather controversial. Tau can be proteolytically processed by the proteasome through an ubiquitin-dependent process, but, since tau is a natively unfolded protein, it was shown that normal tau degradation might not require its preceding ubiquitylation at all (David et al., 2002; Petrucelli et al., 2004; Grune et al., 2010). At the same time, the contribution of tau ubiquitylation in tau pathology has not been completely clarified. Ubiquitin was identified as a protein component of PHFs, NFTs and neurites associated with senile plaques in the brains of patients with AD (Mori et al., 1987; Perry et al., 1987), which appeared to include more ubiquitin compared to normal controls (Riederer et al., 2009). However, given that pre-tangles were not immunostained by an anti-ubiquitin antibody, this suggests that ubiquitin might be linked to fibrillary inclusions only after their formation (Bancher et al., 1991; Garcia-Sierra et al., 2012). Similarly, the ends of neuropil threads, which represent newly formed regions, are characterized by the absence of anti-ubiquitin immunostaining (Iwatsubo et al., 1992). Moreover, abnormal hyperphosphorylation and N-terminal cleavage of tau was shown to precede both the formation as well as the ubiquitylation of tau neurofibrillary inclusions in AD brains (Bancher et al., 1991; Morishima-Kawashima et al., 1993). In contrast, it was reported that both monoubiquitylation and polyubiquitylation contribute to the formation of insoluble protein inclusions present in neurodegenerative diseases (Dickey et al., 2006; Tan et al., 2008) and, as mentioned above, increased aggregation of tau was detected in a cell culture overexpressing CHIP (Petrucelli et al., 2004) implying that ubiquitylation enhances the formation of these aggregates.

Since tau was identified as the ubiquitin-targeted protein in PHFs (Morishima-Kawashima et al., 1993), this has raised questions about the insufficient clearance of pathological fibrillary inclusions of tau. Apart from inaccessibility of the ubiquitylated tau aggregates by the cellular quality control system, the inhibitory binding of PHF-tau to proteasomes is responsible for the proteasomal impairment observed in AD brains (Keck et al., 2003). Additionally, most ubiquitin found in PHFs from AD brains occurs as a monoubiquitylated form, whereas only a small proportion of ubiquitin forms polyubiquitin chains (Morishima-Kawashima et al., 1993), making it difficult to induce UPS-mediated proteolysis of tau aggregates. Another aspect demanding further investigation is the role of the autophagic pathway in removing insoluble tau structures since truncated tau present in AD brains is reported to be preferentially cleared by the autophagic pathway (Rissman et al., 2004; Dolan and Johnson, 2010).

Since impaired tau clearance is widely considered to be a critical factor causing tau accumulation in neurons, many therapeutic approaches targeting tau pathology aim to promote either ubiquitylation or degradation of tau protein. Long-term administration of lithium to murine models of AD-like tauopathies reduces tau lesions primarily by enhancing their ubiquitylation (Nakashima et al., 2005), whereas synthesis of molecules that bring tau and E3 ubiquitin ligases together aims to enhance tau polyubiquitylation and degradation (Chu et al., 2016). Lastly, specific RNA aptamers of USP14, a proteasome-associated deubiquitylating enzyme, inhibited the deubiquitylating activity of this enzyme facilitating the proteasomal degradation of tau *in vitro* (Lee et al., 2015).

## Tau SUMOylation

SUMOylation is another modification in which a small protein is post-translationally attached to the target protein. An ubiquitin-like protein, the small ubiquitin-like modifier (SUMO), is transferred enzymatically to the terminal amino group of lysine side chains of the target protein forming an isopeptide bond in a way similar to ubiquitylation (Figure 2D). Three main SUMO isoforms are expressed in cells, SUMO1, SUMO2, and SUMO3, of which SUMO2 and SUMO3 are more similar to each other than to SUMO1 (Sarge and Park-Sarge, 2009).

Analysis of immunoreactive tau species derived from HEK293 cells expressing tau and different His-tagged SUMO isoforms showed that tau is preferentially monoSUMOylated by SUMO1 and, to a lesser extent, by SUMO2 and SUMO3 (Dorval and Fraser, 2006). Like ubiquitin, SUMO is conjugated by an ATP-dependent enzymatic cascade involving an E1 activating enzyme, an E2 SUMO-conjugating enzyme and an E3-type SUMO ligase. The AOS1-UBA2 complex acts as an E1 activating enzyme, whilst Ubch9 has E2 SUMO-conjugating activity (Desterro et al., 1997; Gong et al., 1999), although there is as yet no direct evidence that they are responsible for tau SUMOylation. Like ubiquitylation, SUMOylation is a reversible process, since specific proteases, called SENPs, can rapidly remove SUMO from their substrates. Although the SENP3 protease was reported to be downregulated in AD tissues (Weeraratna et al., 2007), as with SUMOylation enzymes, no particular proteases have been identified specifically to deSUMOylate tau.

Lysine residues that are targeted for SUMOylation are part of the consensus motif ΨKX(E/D), where Ψ and X represent a hydrophobic residue and any amino acid, respectively, (Dorval and Fraser, 2006). Tau contains two such motifs, VK340SE and AK385TD, however, the examination of SUMOylation at these lysine sites by generating the tau mutants K340R and K385R showed that only K340R displays altered SUMOylation levels indicating that K340, located within the MBD, is the major SUMOylation site on tau (Figure 3D; Dorval and Fraser, 2006). Another study that also used the K340R mutation showed that the effect of tau SUMOylation on HEK293 cells co-transfected with SUMO1 and tau K340R is eliminated, confirming that K340 is indeed a putative SUMOylation site (Luo H. B. et al., 2014). Between valine and alanine, valine displays higher hydrophobicity, potentially explaining why the motif VK340SE might form a more appropriate environment than AK385TD for facilitating the SUMOylation of the included lysine residue.

Tau is available for SUMOylation only after its release from microtubules (Dorval and Fraser, 2006) suggesting that SUMOylation is a post-translational modification that does not target the endogenous tau pool but, consequently, is likely to be involved exclusively in tau pathogenic processes. This agrees with evidence showing that tau hyperphosphorylation, which is the main trigger of tau unbinding from microtubules, facilitates SUMOylation by SUMO1 in HEK293 cells (Luo H. B. et al., 2014). Although hyperphosphorylation possibly precedes SUMOylation, there is increased evidence that tau SUMOylation reciprocally enhances tau hyperphosphorylation at several AD-related sites, such as T231 and S262 (Luo H. B. et al., 2014). SUMOylation also modulates tau ubiquitylation; SUMOylation of hyperphosphorylated tau at K340 inhibits its ubiquitylation and the subsequent proteasome-dependent degradation (Luo H. B. et al., 2014). In contrast, inhibition of the proteasome pathway stimulates tau ubiquitylation, whereas tau SUMOylation is eliminated (Dorval and Fraser, 2006). Possible reasons explaining why tau SUMOylation affects ubiquitylation and *vice versa* is that conjugation of a polypeptide group may inhibit the attachment of another large molecule to the neighboring lysine residues by steric factors or that these two tau modifications compete for the same lysine residues on tau, even though the only putative SUMOylated site, K340, was not identified to be ubiquitylated. SUMOylation could be a factor contributing to the impaired clearance of tau under pathological conditions by upregulating the pool of pathogenic tau in the cytosol and enhancing tau aggregation due to the decreased solubility of SUMOylated tau (Luo H. B. et al., 2014).

Immunohistochemical analysis of brains from APP transgenic mice, a model of AD, revealed that SUMO1 co-localizes with hyperphosphorylated tau in neurites associated with amyloid plaques suggesting that tau SUMOylation is a downstream effect mediated by pathological Aβ amyloid plaques (Takahashi et al., 2008). In agreement with this observation, rat primary hippocampal neurons displayed increasing levels of tau SUMOylation when treated with increasing concentrations of Aβ peptides (Luo H. B. et al., 2014). In the Tg2576 murine model of AD, SUMO1 protein conjugation was elevated both in the cortex and hippocampus (Nistico et al., 2014). Conversely, NFTs found in the brains of AD patients and hyperphosphorylated tau inclusions from mutant tau transgenic mouse brains were both negative for SUMO1 immunoreactivity (Takahashi et al., 2008). In progressive supranuclear palsy brain tissues, SUMO1 colocalizes within perinuclear tau-positive inclusions in oligodendrocytes and labels lysosomes in oligodendrocytes containing tau inclusions, in contrast to those where tau aggregates are absent (Wong et al., 2013). This finding indicates that SUMOylation might be involved in the autophagy-lysosome pathway in tauopathies, but this remains to be elucidated. What is more, increased SUMO1 levels were determined by ELISA in blood plasma derived from both dementia and mild cognitive impairment patients compared to healthy samples suggesting that SUMO1 could serve as an AD biomarker (Cho et al., 2015). Recently, a SUMO1 transgenic mouse model with SUMO1 overexpression in neurons was generated, in which increased levels of SUMO1 eliminated basal synaptic transmission, impaired presynaptic function and reduced spine density, which resulted in learning and memory deficits (Matsuzaki et al., 2015). Since tau is an important SUMO1 target in the cytoplasm and dendritic spines it would be intriguing if hyper-SUMOylation of tau underpinned these deficits, although there are clearly other molecular targets that require follow up.

## Tau glycation

Glycation (or non-enzymatic glycosylation) defines the non-specific reaction in which reducing sugars, especially glucose, are non-enzymatically linked to proteins by condensation of a sugar aldehyde or ketone group with terminal amino groups of lysine side chains (Figure 2E). As a result, glycation depends on both the availability of free lysine amino groups along the polypeptide chain and the concentration of sugar. The glycation products are subject to further changes that lead to the formation of the advanced glycation end products (AGEs) by developing irreversible cross-links with other proteins over a long period of time (Eble et al., 1983). Although, the formation of AGEs does not involve enzymes, there are several enzyme systems that antagonize AGEs production, like the NADPH-dependent aldose reductase and the aldehyde dehydrogenase (Li J. et al., 2012).

Non enzymatic glycation is one of the modifications detected in PHF-tau purified from human AD brains *in vivo*, but not in soluble tau (Figure 3E; Ledesma et al., 1994). It occurs preferentially at the MBD of PHF-tau (Ledesma and Avila, 1995); lysines present at the R3 repeat were initially identified to be glycated *in vitro* (Ledesma et al., 1994), but later studies confirmed immunologically that glycation takes place *in vivo* within the MBD (Ledesma and Avila, 1995). 13 lysine residues, located throughout the polypeptide chain, were also identified as being glycated *in vitro* (Nacharaju et al., 1997); although most of these sites were detected in both 3R and 4R isoforms, K280 and K281 are absent in the case of 3R tau and this difference seems enough to cause slower glycation of 3R compared to 4R isoforms (Nacharaju et al., 1997; Liu et al., 2016). Recently, mass spectrometry analysis revealed 19 novel sites of glycation occurring on recombinant full length tau (Liu et al., 2016).

Since tau has been shown to be glycated within the MBD and glycated tau appears to have reduced affinity for microtubules *in vitro*, it has been suggested that glycation blocks tau-microtubule interactions, thereby assisting tau hyperphosphorylation (Ledesma et al., 1994). The region of tau containing the MBD was shown to participate in its self-association (Ledesma and Avila, 1995), implying that glycation indirectly facilitates the aggregation of tau or stabilizes the aggregated tau species. On the other hand, since glycated PHF-tau has higher tendency for aggregation compared to non-glycated soluble tau, it is likely that glycation stimulates the aggregation of PHFs into more complex structures and stabilizes the assembled formations (Ledesma et al., 1994, 1996; Ko et al., 1999). This suggestion is supported by the observed crosslinking between AGE-modified proteins, which represents an additional factor contributing to the insolubility and resistance against proteolytic degradation that are characteristic of tau aggregates. However, tau glycation enhances, but is not able to trigger, aggregation *in vitro* (Necula and Kuret, 2004). In addition, glycation has different impacts on aggregation propensity depending on the tau isoform that is modified, with the full length tau isoform displaying more extensive aggregation when glycated (Liu et al., 2016).

By immunoelectron microscopy, AGEs were found to colocalize with PHF-tau in NFTs of sporadic AD (Yan et al., 1994). Intracellular AGE-positive deposits were found to correlate positively both with age in normal and AD cases and with the stage of the disease in AD patients (Luth et al., 2005). AGEs may be involved in several processes underlying tau pathogenesis; neurons carrying AGE-positive NFTs also bear intracellular reactive oxygen intermediates (Yan et al., 1994). Moreover, it has been found that the introduction of glycated recombinant tau into neuroblastoma cells is able to generate oxygen free radicals causing neuronal dysfunction by inducing oxidative stress (Yan et al., 1994). Further studies demonstrated that AGEs may be involved in the tau-associated pathogenesis of AD via reactive oxygen intermediates by activating NF-kB-induced transcription, which leads to increased expression of the cytokine IL-6, and by enhancing the synthesis of the amyloid precursor protein, which, as a consequence, promotes the release of the Aβ peptides under stress conditions (Yan et al., 1995). By using reactive carbonyl compounds, which are elevated under conditions of oxidative stress, enhanced formation of AGE-modified tau tangles was observed *in vivo* (Kuhla et al., 2007). AGEs can also cause cellular toxicity through their receptor (RAGE). In a mouse model of AD, tau is colocalized with RAGE in the hippocampus and cortex (Choi et al., 2014) and RAGE has been associated with AGE-induced tau hyperphosphorylation as well as synapse dysfunction and spatial memory impairment in rats (Li X. H. et al., 2012). Lastly, apart from AD, tau-positive inclusions were also AGE-positive in the case of Pick's disease as well as in other neurodegenerative diseases (Sasaki et al., 1998).

## Conclusion

Lysine residues of tau are common targets for different post-translational modifications including acetylation, methylation, ubiquitylation, SUMOylation, and glycation. As a result, it is likely that modification of a target lysine blocks or controls other possible modifications occurring at the same site. Although, the identification of possible sites found to be modified is unlikely yet to be complete, the overlap between each type of post-translational modification in terms of all lysine sites that have been discovered so far is obvious, implying that certain lysine modifications compete for the same site on tau. At the same time, the high content of lysine residues in tau provides sufficient targets to be modified emphasizing the importance of lysine post-translational modification in normal tau biology as well as the mechanisms of misfolding and cellular toxicity elicited by it pathogenic isoform(s). Significantly, the sites modified *in vitro* might differ from those identified in *in vivo* systems, whereas the species, neuronal subtype and pathological state could promote different site-specific modifications as well as certain combinations of modified lysine sites. Therefore, methodical mapping of the lysine residues that can be modified both on endogenous and abnormal tau under different pathological conditions is crucial. The impact of each type of post-translational modification on normal tau or related to pathological states of tau is likely to be site-dependent and the potential for cross-talk of these modifications is high—this fact guides the direction for future scientific research in order to unravel fully the biochemical mechanisms underpinning tau biology.

## Author contributions

CK performed the review, wrote the first draft of the manuscript and prepared publication-ready figures. PP and AG supervised the work, and edited the manuscript into final form. All authors agreed the final version of the manuscript.

### Conflict of interest statement

The authors declare that the research was conducted in the absence of any commercial or financial relationships that could be construed as a potential conflict of interest.

## References

[B1] Babu J. R. Geetha T. Wooten M. W. (2005). Sequestosome 1/p62 shuttles polyubiquitinated tau for proteasomal degradation. J. Neurochem. 94, 192–203. 10.1111/j.1471-4159.2005.03181.x 15953362

[B2] Ballatore C. Lee V. M. Trojanowski J. Q. (2007). Tau-mediated neurodegeneration in Alzheimer's disease and related disorders. Nat. Rev. Neurosci. 8, 663–672. 10.1038/nrn2194 17684513

[B3] Bancher C. Grundke-Iqbal I. Iqbal K. Fried V. A. Smith H. T. Wisniewski H. M. (1991). Abnormal phosphorylation of tau precedes ubiquitination in neurofibrillary pathology of Alzheimer disease. Brain Res. 539, 11–18. 10.1016/0006-8993(91)90681-K 1849776

[B4] Binder L. I. Frankfurter A. Rebhun L. I. (1985). The distribution of tau in the mammalian central nervous system. J. Cell Biol. 101, 1371–1378. 10.1083/jcb.101.4.1371 3930508 PMC2113928

[B5] Blanco-Garcia N. Asensio-Juan E. de la Cruz X. Martinez-Balbas M. A. (2009). Autoacetylation regulates P/CAF nuclear localization. J. Biol. Chem. 284, 1343–1352. 10.1074/jbc.M806075200 19015268

[B6] Carrell R. W. Lomas D. A. (1997). Conformational disease. Lancet 350, 134–138. 10.1016/S0140-6736(97)02073-4 9228977

[B7] Cho S. J. Yun S. M. Lee D. H. Jo C. Ho Park M. Han C.. (2015). Plasma SUMO1 protein is elevated in Alzheimer's disease. J. Alzheimers. Dis. 47, 639–643. 10.3233/JAD-150103 26401699

[B8] Choi B. R. Cho W. H. Kim J. Lee H. J. Chung C. Jeon W. K.. (2014). Increased expression of the receptor for advanced glycation end products in neurons and astrocytes in a triple transgenic mouse model of Alzheimer's disease. Exp. Mol. Med. 46:e75. 10.1038/emm.2013.147. 24503708 PMC3909893

[B9] Chu T. T. Gao N. Li Q. Q. Chen P. G. Yang X. F. Chen Y. X.. (2016). Specific knockdown of endogenous tau protein by peptide-directed ubiquitin-proteasome degradation. Cell Chem. Biol. 23, 453–461. 10.1016/j.chembiol.2016.02.016 27105281

[B10] Cohen T. J. Constance B. H. Hwang A. W. James M. Yuan C. X. (2016). Intrinsic tau acetylation is coupled to auto-proteolytic tau fragmentation. PLoS ONE 11:e0158470. 10.1371/journal.pone.0158470 27383765 PMC4934699

[B11] Cohen T. J. Friedmann D. Hwang A. W. Marmorstein R. Lee V. M. (2013). The microtubule-associated tau protein has intrinsic acetyltransferase activity. Nat. Struct. Mol. Biol. 20, 756–762. 10.1038/nsmb.2555 23624859 PMC3827724

[B12] Cohen T. J. Guo J. L. Hurtado D. E. Kwong L. K. I. Mills Trojanowski J. Q.. (2011). The acetylation of tau inhibits its function and promotes pathological tau aggregation. Nat. Commun. 2:252. 10.1038/ncomms1255. 21427723 PMC3120096

[B13] Cook C. Carlomagno Y. Gendron T. F. Dunmore J. Scheffel K. Stetler C.. (2014). Acetylation of the KXGS motifs in tau is a critical determinant in modulation of tau aggregation and clearance. Hum. Mol. Genet. 23, 104–116. 10.1093/hmg/ddt402 23962722 PMC3857946

[B14] Cook C. Gendron T. F. Scheffel K. Carlomagno Y. Dunmore J. DeTure M.. (2012). Loss of HDAC6, a novel CHIP substrate, alleviates abnormal tau accumulation. Hum. Mol. Genet. 21, 2936–2945. 10.1093/hmg/dds125 22492994 PMC3373241

[B15] Cripps D. Thomas S. N. Jeng Y. Yang F. Davies P. Yang A. J. (2006). Alzheimer disease-specific conformation of hyperphosphorylated paired helical filament-Tau is polyubiquitinated through Lys-48, Lys-11, and Lys-6 ubiquitin conjugation. J. Biol. Chem. 281, 10825–10838. 10.1074/jbc.M512786200 16443603

[B16] David D. C. Layfield R. Serpell L. Narain Y. Goedert M. Spillantini M. G. (2002). Proteasomal degradation of tau protein. J. Neurochem. 83, 176–185. 10.1046/j.1471-4159.2002.01137.x 12358741

[B17] Desterro J. M. Thomson J. Hay R. T. (1997). Ubch9 conjugates SUMO but not ubiquitin. FEBS Lett. 417, 297–300. 10.1016/S0014-5793(97)01305-7 9409737

[B18] Dickey C. A. Koren J. Zhang Y. J. Xu Y. F. Jinwal U. K. Birnbaum M. J.. (2008). Akt and CHIP coregulate tau degradation through coordinated interactions. Proc. Natl. Acad. Sci. U.S.A. 105, 3622–3627. 10.1073/pnas.0709180105 18292230 PMC2265134

[B19] Dickey C. A. Yue M. Lin W. L. Dickson D. W. Dunmore J. H. Lee W. C. (2006). Deletion of the ubiquitin ligase CHIP leads to the accumulation, but not the aggregation, of both endogenous phospho- and caspase-3-cleaved tau species. J. Neurosci. 26, 6985–6996. 10.1523/JNEUROSCI.0746-06.2006 16807328 PMC6673930

[B20] Dickson D. W. Kouri N. Murray M. E. Josephs K. A. (2011). Neuropathology of frontotemporal lobar degeneration-tau (FTLD-tau). J. Mol. Neurosci. 45, 384–389. 10.1007/s12031-011-9589-0 21720721 PMC3208128

[B21] Ding H. Dolan P. J. Johnson G. V. (2008). Histone deacetylase 6 interacts with the microtubule-associated protein tau. J. Neurochem. 106, 2119–2130. 10.1111/j.1471-4159.2008.05564.x 18636984 PMC2574575

[B22] Dolan P. J. Johnson G. V. (2010). A caspase cleaved form of tau is preferentially degraded through the autophagy pathway. J. Biol. Chem. 285, 21978–21987. 10.1074/jbc.M110.110940 20466727 PMC2903354

[B23] Dorval V. Fraser P. E. (2006). Small ubiquitin-like modifier (SUMO) modification of natively unfolded proteins tau and alpha-synuclein. J. Biol. Chem. 281, 9919–9924. 10.1074/jbc.M510127200 16464864

[B24] Eble A. S. Thorpe S. R. Baynes J. W. (1983). Nonenzymatic glucosylation and glucose-dependent cross-linking of protein. J. Biol. Chem. 258, 9406–9412. 6409904

[B25] Ferrer I. Lopez-Gonzalez I. Carmona M. Arregui L. Dalfo E. Torrejon-Escribano B.. (2014). Glial and neuronal tau pathology in tauopathies: characterization of disease-specific phenotypes and tau pathology progression. J. Neuropathol. Exp. Neurol. 73, 81–97. 10.1097/NEN.0000000000000030 24335532

[B26] Flach K. Ramminger E. Hilbrich I. Arsalan-Werner A. Albrecht F. Herrmann L.. (2014). Axotrophin/MARCH7 acts as an E3 ubiquitin ligase and ubiquitinates tau protein *in vitro* impairing microtubule binding. Biochim. Biophys. Acta 1842, 1527–1538. 10.1016/j.bbadis.2014.05.029 24905733 PMC4311138

[B27] Funk K. E. Thomas S. N. Schafer K. N. Cooper G. L. Liao Z. Clark D. J.. (2014). Lysine methylation is an endogenous post-translational modification of tau protein in human brain and a modulator of aggregation propensity. Biochem. J. 462, 77–88. 10.1042/BJ20140372 24869773 PMC4292886

[B28] Garcia-Sierra F. Jarero-Basulto J. J. Kristofikova Z. Majer E. Binder L. I. Ripova D. (2012). Ubiquitin is associated with early truncation of tau protein at aspartic acid(421) during the maturation of neurofibrillary tangles in Alzheimer's disease. Brain Pathol. 22, 240–250. 10.1111/j.1750-3639.2011.00525.x 21919991 PMC8029281

[B29] Gong L. Li B. Millas S. Yeh E. T. (1999). Molecular cloning and characterization of human AOS1 and UBA2, components of the sentrin-activating enzyme complex. FEBS Lett. 448, 185–189. 10.1016/S0014-5793(99)00367-1 10217437

[B30] Gorsky M. K. Burnouf S. Dols J. Mandelkow E. Partridge L. (2016). Acetylation mimic of lysine 280 exacerbates human tau neurotoxicity *in vivo*. Sci. Rep. 6:22685. 10.1038/srep22685 26940749 PMC4778021

[B31] Gratuze M. Cisbani G. Cicchetti F. Planel E. (2016). Is Huntington's disease a tauopathy? Brain 139(Pt 4), 1014–1025. 10.1093/brain/aww021 26969684

[B32] Grinberg L. T. Wang X. Wang C. Sohn P. D. Theofilas P. Sidhu M.. (2013). Argyrophilic grain disease differs from other tauopathies by lacking tau acetylation. Acta Neuropathol. 125, 581–593. 10.1007/s00401-013-1080-2 23371364 PMC3692283

[B33] Grundke-Iqbal I. Iqbal K. Tung Y. C. Quinlan M. Wisniewski H. M. Binder L. I. (1986). Abnormal phosphorylation of the microtubule-associated protein tau (tau) in Alzheimer cytoskeletal pathology. Proc. Natl. Acad. Sci. U.S.A. 83, 4913–4917. 10.1073/pnas.83.13.4913 3088567 PMC323854

[B34] Grune T. Botzen D. Engels M. Voss P. Kaiser B. Jung T.. (2010). Tau protein degradation is catalyzed by the ATP/ubiquitin-independent 20S proteasome under normal cell conditions. Arch. Biochem. Biophys. 500, 181–188. 10.1016/j.abb.2010.05.008 20478262 PMC2904402

[B35] Hernandez F. Avila J. (2007). Tauopathies. Cell. Mol. Life Sci. 64, 2219–2233. 10.1007/s00018-007-7220-x 17604998 PMC11136052

[B36] Hofmann R. M. Pickart C. M. (2001). *In vitro* assembly and recognition of Lys-63 polyubiquitin chains. J. Biol. Chem. 276, 27936–27943. 10.1074/jbc.M103378200 11369780

[B37] Hwang A. W. Trzeciakiewicz H. Friedmann D. Yuan C. X. Marmorstein R. Lee V. M.. (2016). Conserved lysine acetylation within the microtubule-binding domain regulates map2/tau family members. PLoS ONE 11:e0168913. 10.1371/journal.pone.0168913 28002468 PMC5176320

[B38] Ikeda F. Dikic I. (2008). Atypical ubiquitin chains: new molecular signals. ‘Protein Modifications: Beyond the Usual Suspects’ review series. EMBO Rep. 9, 536–542. 10.1038/embor.2008.93. 18516089 PMC2427391

[B39] Irwin D. J. Cohen T. J. Grossman M. Arnold S. E. McCarty-Wood E. Van Deerlin V. M.. (2013). Acetylated tau neuropathology in sporadic and hereditary tauopathies. Am. J. Pathol. 183, 344–351. 10.1016/j.ajpath.2013.04.025 23885714 PMC3730769

[B40] Irwin D. J. Cohen T. J. Grossman M. Arnold S. E. Xie S. X. Lee V. M.. (2012). Acetylated tau, a novel pathological signature in Alzheimer's disease and other tauopathies. Brain 135(Pt. 3), 807–818. 10.1093/brain/aws013 22366796 PMC3286338

[B41] Iwata A. Riley B. E. Johnston J. A. Kopito R. R. (2005). HDAC6 and microtubules are required for autophagic degradation of aggregated huntingtin. J. Biol. Chem. 280, 40282–40292. 10.1074/jbc.M508786200 16192271

[B42] Iwatsubo T. Hasegawa M. Esaki Y. Ihara Y. (1992). Lack of ubiquitin immunoreactivities at both ends of neuropil threads. Possible bidirectional growth of neuropil threads. Am. J. Pathol. 140, 277–282. 1310831 PMC1886416

[B43] Julien C. Tremblay C. Emond V. Lebbadi M. Salem N. Jr. Bennett D. A.. (2009). Sirtuin 1 reduction parallels the accumulation of tau in Alzheimer disease. J. Neuropathol. Exp. Neurol. 68, 48–58. 10.1097/NEN.0b013e3181922348 19104446 PMC2813570

[B44] Kamah A. Huvent I. Cantrelle F. X. Qi H. Lippens G. Landrieu I.. (2014). Nuclear magnetic resonance analysis of the acetylation pattern of the neuronal Tau protein. Biochemistry 53, 3020–3032. 10.1021/bi500006v 24708343

[B45] Keck S. Nitsch R. Grune T. Ullrich O. (2003). Proteasome inhibition by paired helical filament-tau in brains of patients with Alzheimer's disease. J. Neurochem. 85, 115–122. 10.1046/j.1471-4159.2003.01642.x 12641733

[B46] Ko L. W. Ko E. C. Nacharaju P. Liu W. K. Chang E. Kenessey A.. (1999). An immunochemical study on tau glycation in paired helical filaments. Brain Res. 830, 301–313. 10.1016/S0006-8993(99)01415-8 10366687

[B47] Kovacs G. G. (2016). Molecular pathological classification of neurodegenerative diseases: turning towards precision medicine. Int. J. Mol. Sci. 17:189. 10.3390/ijms17020189 26848654 PMC4783923

[B48] Kuhla B. Haase C. Flach K. Luth H. J. Arendt T. Munch G. (2007). Effect of pseudophosphorylation and cross-linking by lipid peroxidation and advanced glycation end product precursors on tau aggregation and filament formation. J. Biol. Chem. 282, 6984–6991. 10.1074/jbc.M609521200 17082178

[B49] Lasagna-Reeves C. A. Castillo-Carranza D. L. Guerrero-Muoz M. J. Jackson G. R. Kayed R. (2010). Preparation and characterization of neurotoxic tau oligomers. Biochemistry 49, 10039–10041. 10.1021/bi1016233 21047142

[B50] Ledesma M. D. Bonay P. Avila J. (1995). Tau protein from Alzheimer's disease patients is glycated at its tubulin-binding domain. J. Neurochem. 65, 1658–1664. 7561862 10.1046/j.1471-4159.1995.65041658.x

[B51] Ledesma M. D. Bonay P. Colaco C. Avila J. (1994). Analysis of microtubule-associated protein tau glycation in paired helical filaments. J. Biol. Chem. 269, 21614–21619. 8063802

[B52] Ledesma M. D. Medina M. Avila J. (1996). The *in vitro* formation of recombinant tau polymers: effect of phosphorylation and glycation. Mol. Chem. Neuropathol. 27, 249–258. 10.1007/BF02815107 9147411

[B53] Lee G. Cowan N. Kirschner M. (1988). The primary structure and heterogeneity of tau protein from mouse brain. Science 239, 285–288. 10.1126/science.3122323 3122323

[B54] Lee G. Neve R. L. Kosik K. S. (1989). The microtubule binding domain of tau protein. Neuron 2, 1615–1624. 10.1016/0896-6273(89)90050-0 2516729

[B55] Lee J. H. Shin S. K. Jiang Y. Choi W. H. Hong C. Kim D. E.. (2015). Facilitated tau degradation by usp14 aptamers via enhanced proteasome activity. Sci. Rep. 5:10757. 10.1038/srep10757 26041011 PMC4455164

[B56] Lei P. Ayton S. Finkelstein D. I. Spoerri L. Ciccotosto G. D. Wright D. K.. (2012). Tau deficiency induces parkinsonism with dementia by impairing APP-mediated iron export. Nat. Med. 18, 291–295. 10.1038/nm.2613 22286308

[B57] Li J. Liu D. Sun L. Lu Y. Zhang Z. (2012). Advanced glycation end products and neurodegenerative diseases: mechanisms and perspective. J. Neurol. Sci. 317, 1–5. 10.1016/j.jns.2012.02.018 22410257

[B58] Li X. H. Lv B. L. Xie J. Z. Liu J. Zhou X. W. Wang J. Z. (2012). AGEs induce Alzheimer-like tau pathology and memory deficit via RAGE-mediated GSK-3 activation. Neurobiol. Aging 33, 1400–1410. 10.1016/j.neurobiolaging.2011.02.003 21450369

[B59] Liu K. Liu Y. Li L. Qin P. Iqbal J. Deng Y.. (2016). Glycation alter the process of tau phosphorylation to change tau isoforms aggregation property. Biochim. Biophys. Acta 1862, 192–201. 10.1016/j.bbadis.2015.12.002 26655600

[B60] Loomis P. A. Howard T. H. Castleberry R. P. Binder L. I. (1990). Identification of nuclear tau isoforms in human neuroblastoma cells. Proc. Natl. Acad. Sci. U.S.A. 87, 8422–8426. 10.1073/pnas.87.21.8422 1700432 PMC54968

[B61] Luo H. B. Xia Y. Y. Shu X. J. Liu Z. C. Feng Y. Liu X. H. (2014). SUMOylation at K340 inhibits tau degradation through deregulating it s phosphorylation and ubiquitination. Proc. Natl. Acad. Sci. U.S.A. 111, 16586–16591. 10.1073/pnas.1417548111 25378699 PMC4246270

[B62] Luo Y. Ma B. Nussinov R. Wei G. (2014). Structural insight into tau protein's paradox of intrinsically disordered behavior, self-acetylation activity, and aggregation. J. Phys. Chem. Lett. 5, 3026–3031. 10.1021/jz501457f 25206938 PMC4154703

[B63] Luth H. J. Ogunlade V. Kuhla B. Kientsch-Engel R. Stahl W. J. Webster J.. (2005). Age- and stage-dependent accumulation of advanced glycation end products in intracellular deposits in normal and Alzheimer's disease brains. Cereb. Cortex 15, 211–220. 10.1093/cercor/bhh123 15238435

[B64] Mandelkow E. M. Biernat J. Drewes G. Gustke N. Trinczek B. Mandelkow E. (1995). Tau domains, phosphorylation, and interactions with microtubules. Neurobiol. Aging 16, 355–362. discussion: 362–363. 10.1016/0197-4580(95)00025-A 7566345

[B65] Martin L. Latypova X. Terro F. (2011). Post-translational modifications of tau protein: implications for Alzheimer's disease. Neurochem. Int. 58, 458–471. 10.1016/j.neuint.2010.12.023 21215781

[B66] Matsuzaki S. Lee L. Knock E. Srikumar T. Sakurai M. Hazrati L. N.. (2015). SUMO1 affects synaptic function, spine density and memory. Sci. Rep. 5:10730. 10.1038/srep10730. 26022678 PMC4650663

[B67] Michishita E. Park J. Y. Burneskis J. M. Barrett J. C. Horikawa I. (2005). Evolutionarily conserved and nonconserved cellular localizations and functions of human SIRT proteins. Mol. Biol. Cell 16, 4623–4635. 10.1091/mbc.E05-01-0033 16079181 PMC1237069

[B68] Migheli A. Butler M. Brown K. Shelanski M. L. (1988). Light and electron microscope localization of the microtubule-associated tau protein in rat brain. J. Neurosci. 8, 1846–1851. 3133453 10.1523/JNEUROSCI.08-06-01846.1988 PMC6569338

[B69] Min S. W. Chen X. Tracy T. E. Li Y. Zhou Y. Wang C.. (2015). Critical role of acetylation in tau-mediated neurodegeneration and cognitive deficits. Nat. Med. 21, 1154–1162. 10.1038/nm.3951 26390242 PMC4598295

[B70] Min S. W. Cho S. H. Zhou Y. Schroeder S. Haroutunian V. Seeley W. W.. (2010). Acetylation of tau inhibits its degradation and contributes to tauopathy. Neuron 67, 953–966. 10.1016/j.neuron.2010.08.044 20869593 PMC3035103

[B71] Mori H. Kondo J. Ihara Y. (1987). Ubiquitin is a component of paired helical filaments in Alzheimer's disease. Science 235, 1641–1644. 10.1126/science.3029875 3029875

[B72] Morishima-Kawashima M. Hasegawa M. Takio K. Suzuki M. Titani K. Ihara Y. (1993). Ubiquitin is conjugated with amino-terminally processed tau in paired helical filaments. Neuron 10, 1151–1160. 10.1016/0896-6273(93)90063-W 8391280

[B73] Morris M. Knudsen G. M. Maeda S. Trinidad J. C. Ioanoviciu A. Burlingame A. L.. (2015). Tau post-translational modifications in wild-type and human amyloid precursor protein transgenic mice. Nat. Neurosci. 18, 1183–1189. 10.1038/nn.4067 26192747 PMC8049446

[B74] Mukhopadhyay D. Riezman H. (2007). Proteasome-independent functions of ubiquitin in endocytosis and signaling. Science 315, 201–205. 10.1126/science.1127085 17218518

[B75] Mukrasch M. D. Bibow S. Korukottu J. Jeganathan S. Biernat J. Griesinger C.. (2009). Structural polymorphism of 441-residue tau at single residue resolution. PLoS Biol. 7:e34. 10.1371/journal.pbio.1000034 19226187 PMC2642882

[B76] Nacharaju P. Ko L. Yen S. H. (1997). Characterization of *in vitro* glycation sites of tau. J. Neurochem. 69, 1709–1719. 10.1046/j.1471-4159.1997.69041709.x 9326300

[B77] Nakashima H. Ishihara T. Suguimoto P. Yokota O. Oshima E. Kugo A.. (2005). Chronic lithium treatment decreases tau lesions by promoting ubiquitination in a mouse model of tauopathies. Acta Neuropathol. 110, 547–556. 10.1007/s00401-005-1087-4 16228182

[B78] Nathan J. A. Kim H. T. Ting L. Gygi S. Goldberg A. L. (2013). Why do cellular proteins linked to K63-polyubiquitin chains not associate with proteasomes? EMBO J. 32, 552–565. 10.1038/emboj.2012.354 23314748 PMC3579138

[B79] Necula M. Kuret J. (2004). Pseudophosphorylation and glycation of tau protein enhance but do not trigger fibrillization *in vitro*. J. Biol. Chem. 279, 49694–49703. 10.1074/jbc.M405527200 15364924

[B80] Neve R. L. Harris P. Kosik K. S. Kurnit D. M. Donlon T. A. (1986). Identification of cDNA clones for the human microtubule-associated protein tau and chromosomal localization of the genes for tau and microtubule-associated protein 2. Brain Res. 387, 271–280. 10.1016/0169-328X(86)90033-1 3103857

[B81] Nistico R. Ferraina C. Marconi V. Blandini F. Negri L. Egebjerg J.. (2014). Age-related changes of protein SUMOylation balance in the AbetaPP Tg2576 mouse model of Alzheimer's disease. Front. Pharmacol. 5:63. 10.3389/fphar.2014.00063. 24778618 PMC3985012

[B82] Pallas-Bazarra N. Jurado-Arjona J. Navarrete M. Esteban J. A. Hernandez F. Avila J.. (2016). Novel function of Tau in regulating the effects of external stimuli on adult hippocampal neurogenesis. EMBO J. 35, 1417–1436. 10.15252/embj.201593518 27198172 PMC4876034

[B83] Perry G. Friedman R. Shaw G. Chau V. (1987). Ubiquitin is detected in neurofibrillary tangles and senile plaque neurites of Alzheimer disease brains. Proc. Natl. Acad. Sci. U.S.A. 84, 3033–3036. 10.1073/pnas.84.9.3033 3033674 PMC304795

[B84] Petrucelli L. Dickson D. Kehoe K. Taylor J. Snyder H. Grover A.. (2004). CHIP and Hsp70 regulate tau ubiquitination, degradation and aggregation. Hum. Mol. Genet. 13, 703–714. 10.1093/hmg/ddh083 14962978

[B85] Riederer I. M. Schiffrin M. Kovari E. Bouras C. Riederer B. M. (2009). Ubiquitination and cysteine nitrosylation during aging and Alzheimer's disease. Brain Res. Bull. 80, 233–241. 10.1016/j.brainresbull.2009.04.018 19427371

[B86] Rissman R. A. Poon W. W. Blurton-Jones M. Oddo S. Torp R. Vitek M.. (2004). Caspase-cleavage of tau is an early event in Alzheimer disease tangle pathology. J. Clin. Invest. 114, 121–130. 10.1172/JCI200420640 15232619 PMC437967

[B87] Sahara N. Murayama M. Mizoroki T. Urushitani M. Imai Y. Takahashi R.. (2005). *In vivo* evidence of CHIP up-regulation attenuating tau aggregation. J. Neurochem. 94, 1254–1263. 10.1111/j.1471-4159.2005.03272.x 16111477

[B88] Sarge K. D. Park-Sarge O. K. (2009). Sumoylation and human disease pathogenesis. Trends Biochem. Sci. 34, 200–205. 10.1016/j.tibs.2009.01.004 19282183 PMC2974900

[B89] Sasaki N. Fukatsu R. Tsuzuki K. Hayashi Y. Yoshida T. Fujii N.. (1998). Advanced glycation end products in Alzheimer's disease and other neurodegenerative diseases. Am. J. Pathol. 153, 1149–1155. 10.1016/S0002-9440(10)65659-3 9777946 PMC1853056

[B90] Schweers O. Schonbrunn-Hanebeck E. Marx A. Mandelkow E. (1994). Structural studies of tau protein and Alzheimer paired helical filaments show no evidence for beta-structure. J. Biol. Chem. 269, 24290–24297. 7929085

[B91] Shimura H. Schwartz D. Gygi S. P. Kosik K. S. (2004). CHIP-Hsc70 complex ubiquitinates phosphorylated tau and enhances cell survival. J. Biol. Chem. 279, 4869–4876. 10.1074/jbc.M305838200 14612456

[B92] Sinha S. Lopes D. H. Du Z. Pang E. S. Shanmugam A. Lomakin A.. (2011). Lysine-specific molecular tweezers are broad-spectrum inhibitors of assembly and toxicity of amyloid proteins. J. Am. Chem. Soc. 133, 16958–16969. 10.1021/ja206279b 21916458 PMC3210512

[B93] Sohn P. D. Tracy T. E. Son H. I. Zhou Y. Leite R. E. Miller B. L.. (2016). Acetylated tau destabilizes the cytoskeleton in the axon initial segment and is mislocalized to the somatodendritic compartment. Mol. Neurodegener. 11:47. 10.1186/s13024-016-0109-0 27356871 PMC4928318

[B94] Spillantini M. G. Goedert M. Crowther R. A. Murrell J. R. Farlow M. R. Ghetti B. (1997). Familial multiple system tauopathy with presenile dementia: a disease with abundant neuronal and glial tau filaments. Proc. Natl. Acad. Sci. U.S.A. 94, 4113–4118. 10.1073/pnas.94.8.4113 9108114 PMC20577

[B95] Takahashi K. Ishida M. Komano H. Takahashi H. (2008). SUMO-1 immunoreactivity co-localizes with phospho-tau in APP transgenic mice but not in mutant Tau transgenic mice. Neurosci. Lett. 441, 90–93. 10.1016/j.neulet.2008.06.012 18586401

[B96] Tan J. M. Wong E. S. Kirkpatrick D. S. Pletnikova O. Ko H. S. Tay S.. (2008). Lysine 63-linked ubiquitination promotes the formation and autophagic clearance of protein inclusions associated with neurodegenerative diseases. Hum. Mol. Genet. 17, 431–439. 10.1093/hmg/ddm320 17981811

[B97] Thomas S. N. Funk K. E. Wan Y. Liao Z. Davies P. Kuret J.. (2012). Dual modification of Alzheimer's disease PHF-tau protein by lysine methylation and ubiquitylation: a mass spectrometry approach. Acta Neuropathol. 123, 105–117. 10.1007/s00401-011-0893-0 22033876 PMC3249157

[B98] Tracy T. E. Sohn P. D. Minami S. S. Wang C. Min S. W. Li Y.. (2016). Acetylated tau obstructs KIBRA-mediated signaling in synaptic plasticity and promotes tauopathy-related memory loss. Neuron 90, 245–260. 10.1016/j.neuron.2016.03.005 27041503 PMC4859346

[B99] Verkhratsky A. Parpura V. Pekna M. Pekny M. Sofroniew M. (2014). Glia in the pathogenesis of neurodegenerative diseases. Biochem. Soc. Trans. 42, 1291–1301. 10.1042/BST20140107 25233406

[B100] Wang P. Joberty G. Buist A. Vanoosthuyse A. Stancu I. C. Vasconcelos B.. (2017). Tau interactome mapping based identification of Otub1 as tau deubiquitinase involved in accumulation of pathological tau forms *in vitro* and *in vivo*. Acta Neuropathol. 133, 731–749. 10.1007/s00401-016-1663-9 28083634 PMC5390007

[B101] Weeraratna A. T. Kalehua A. Deleon I. Bertak D. Maher G. Wade M. S.. (2007). Alterations in immunological and neurological gene expression patterns in Alzheimer's disease tissues. Exp. Cell Res. 313, 450–461. 10.1016/j.yexcr.2006.10.028 17188679 PMC2565515

[B102] West L. E. Gozani O. (2011). Regulation of p53 function by lysine methylation. Epigenomics 3, 361–369. 10.2217/epi.11.21 21826189 PMC3151012

[B103] Williamson R. Scales T. Clark B. R. Gibb G. Reynolds C. H. Kellie S.. (2002). Rapid tyrosine phosphorylation of neuronal proteins including tau and focal adhesion kinase in response to amyloid-beta peptide exposure: involvement of Src family protein kinases. J. Neurosci. 22, 10–20. Available online at: http://www.jneurosci.org/content/22/1/10.long 11756483 10.1523/JNEUROSCI.22-01-00010.2002 PMC6757621

[B104] Wong M. B. Goodwin J. Norazit A. Meedeniya A. C. Richter-Landsberg C. Gai W.. (2013). SUMO-1 is associated with a subset of lysosomes in glial protein aggregate diseases. Neurotox. Res. 23, 1–21. 10.1007/s12640-012-9358-z 23229893

[B105] Xu Z. Kohli E. Devlin K. I. Bold M. Nix J. C. Misra S. (2008). Interactions between the quality control ubiquitin ligase CHIP and ubiquitin conjugating enzymes. BMC Struct. Biol. 8:26. 10.1186/1472-6807-8-26 18485199 PMC2396629

[B106] Yan S. D. Chen X. Schmidt A. M. Brett J. Godman G. Zou Y. S.. (1994). Glycated tau protein in Alzheimer disease: a mechanism for induction of oxidant stress. Proc. Natl. Acad. Sci. U.S.A. 91, 7787–7791. 10.1073/pnas.91.16.7787 8052661 PMC44487

[B107] Yan S. D. Yan S. F. Chen X. Fu J. Chen M. Kuppusamy P.. (1995). Non-enzymatically glycated tau in Alzheimer's disease induces neuronal oxidant stress resulting in cytokine gene expression and release of amyloid beta-peptide. Nat. Med. 1, 693–699. 10.1038/nm0795-693 7585153

[B108] Yoshida M. (2006). Cellular tau pathology and immunohistochemical study of tau isoforms in sporadic tauopathies. Neuropathology 26, 457–470. 10.1111/j.1440-1789.2006.00743.x 17080726

[B109] Zhang Y. J. Xu Y. F. Liu X. H. Li D. Yin J. Liu Y. H.. (2008). Carboxyl terminus of heat-shock cognate 70-interacting protein degrades tau regardless its phosphorylation status without affecting the spatial memory of the rats. J. Neural. Transm. 115, 483–491. 10.1007/s00702-007-0857-7 18301957

